# Multimodal imaging in a case of quadricuspid aortic valve regurgitation and stenosis: diagnostic challenges before transfemoral aortic valve replacement implantation

**DOI:** 10.1093/ehjimp/qyaf087

**Published:** 2025-07-08

**Authors:** Louise Sakowski, Vladyslav Kavalerchyk

**Affiliations:** Department of Internal Medicine, Piedmont Macon Medical Center, 350 Hospital Dr, Macon, GA 31217, USA; Department of Cardiology, Helios Hospital Schwerin, Wismarsche Str. 393-397, Schwerin 19055, Germany

**Keywords:** quadricuspid aortic valve, echocardiopgraphy, cardiac CT, TAVR

An 83-year-old patient was evaluated for a transfemoral aortic valve replacement (TAVR) due to severe aortic valve regurgitation and stenosis. The patient exhibited shortness of breath (New York Heart Association class III) and dizziness but denied chest pain, syncope, fever, or chills. Clinical examination revealed a crescendo–decrescendo systolic murmur most audible over the right second intercostal space. Electrocardiogram studies showed atrial fibrillation and left axis deviation. The initial pre-procedural transthoracic echocardiogram showed a quadricuspid aortic valve with severe aortic stenosis and severe aortic valve regurgitation (*Figure 1A* and *1B*). However, transoesophageal echocardiogram, while confirming severe aortic stenosis and regurgitation, could only identify a tricuspid aortic valve, creating uncertainty about the number of leaflets (*Figure 1C*). To confirm the presence of a quadricuspid valve and ensure accurate sizing for the TAVR prosthesis, a cardiac computed tomography (CT) scan was performed (*Figure 2A* and *2B*). The CT scan demonstrated a severely calcified quadricuspid aortic valve, classified as Hurwitz and Roberts class A with four equal leaflets. The patient underwent TAVR with a 26 mm Edwards prosthesis (*Figure 4*). During the procedure, the patient required electrocardioversion twice due to peri-procedural tachyarrhythmias. Otherwise, the procedure was unremarkable. Post-procedural echocardiography confirmed the proper placement of the Edwards valve without any paravalvular leakage (*Figure 3A* and *3B*). The patient experienced no complications and was discharged home 4 days after the procedure.

**Figure 1:**
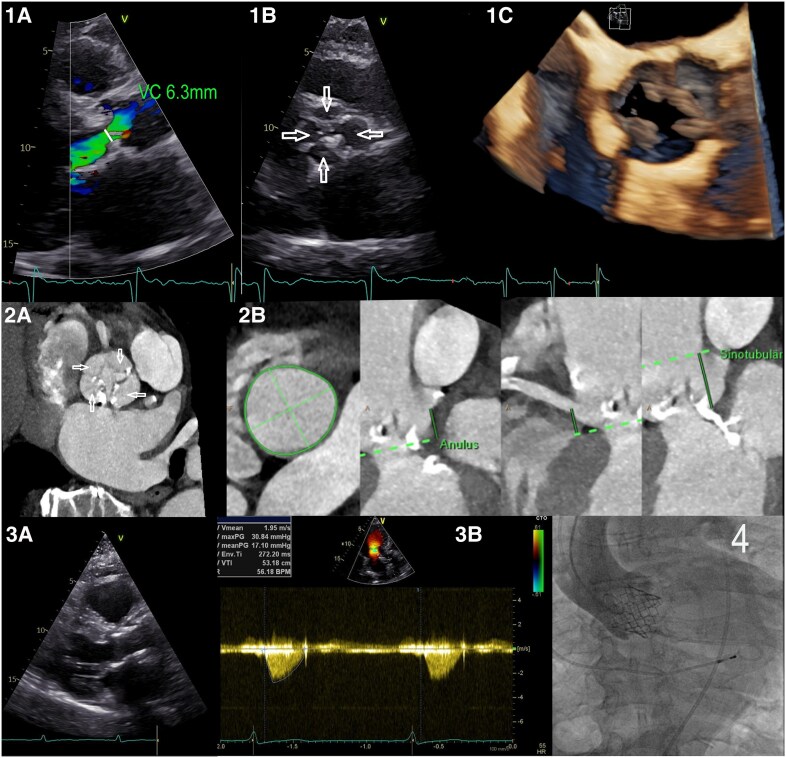
Echocardiographic and CT images. (*1A*) Transthoracic echocardiogram parasternal long-axis view demonstrating aortic valve regurgitation with vena contracta measuring 6.3 mm (white line). (*1B*) Transthoracic echocardiogram parasternal short-axis view showing four leaflets of the aortic valve (white arrows). (*1C*) Real-time 3D transoesophageal echocardiogram view depicting the aortic valve with three leaflets. Figure 2: CT images of the heart. (*2A*) CT scan of the heart in short-axis view illustrating the presence of four leaflets (white arrows). (*2B*) CT scan demonstrating the distance from the ostium to coronary arteries during pre-procedural planning. Figure 3: Echocardiographic views post-TAVR. (*3A*) Transthoracic echocardiogram parasternal long-axis view showing optimal positioning of TAVR post-implantation. (*3B*) Transthoracic echocardiogram apical long-axis view with Doppler ultrasound confirming favourable results following TAVR procedure. Figure 4: Fluoroscopy. Fluoroscopic image depicting successful TAVR implantation.

**Consent:** Informed consent was achieved from the patient in written and oral form to write this case study.

**Funding:** This research was not supported by any sponsor or funder.

**Data availability:** No new data were generated or analysed in support of this research.

## Lead author biography


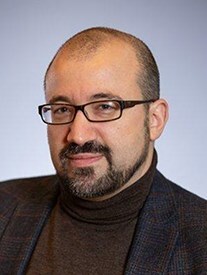


Dr Vladyslav Kavalerchyk is an experienced physician specializing in functional diagnostics at the Helios Kliniken Schwerin, with extensive expertise in echocardiography. He leads the echocardiography lab since 2017 and holds advanced qualifications in transthoracic, transoesophageal, stress, and contrast echocardiography. As a member of the EACVI Web & Communication Committee and a certified trainer (DEGUM II), he plays an active role in advancing echocardiographic education and clinical research.

